# Crystal structure of *N*-ethyl-2-(1,2,3,4-tetra­hydro­naphthalen-1-yl­idene)hydrazinecarbo­thio­amide

**DOI:** 10.1107/S2056989017001311

**Published:** 2017-01-31

**Authors:** Adriano Bof de Oliveira, Johannes Beck, Christian Landvogt, Renan Lira de Farias, Bárbara Regina Santos Feitoza

**Affiliations:** a Universidade Federal de Sergipe (UFS), Departamento de Química, São Cristóvão, Brazil; b Rheinische Friedrich-Wilhelms-Universität Bonn, Institut für Anorganische Chemie, Bonn, Germany; c Universidade Estadual Paulista (UNESP), Instituto de Química, Araraquara, Brazil

**Keywords:** crystal structure, tetra­lone thio­semicarbazone derivative, Hirshfeld surface calculation

## Abstract

There are two crystallographically independent mol­ecules in the asymmetric unit of the title compound, C_13_H_17_N_3_S, one of them being disordered over the methyl group [site-occupancy ratio = 0.705 (5):0.295 (5)]. The maximum r.m.s. deviations from the mean plane of the non-H atoms for the tetra­lone fragments amount to 0.4572 (17) and 0.4558 (15) Å. The N—N—C—N fragments are not planar and torsion angles are −9.4 (2) and 8.3 (2)°. In the crystal, the mol­ecules are linked by weak N—H⋯S inter­actions into chains along [100] with graph-set motif *C*(4) and connected by weak N—H⋯S and C—H⋯S inter­actions, forming *R*_2_^1^(10) rings. The Hirshfeld surface analysis indicates that the most important contributions for the crystal packing are the H⋯H (64.20%), H⋯S (12.60%) and H⋯C (12.00%) inter­actions. The crystal packing resembles a herringbone arrangement when viewed along [001].

## Abstract 2

There are two mol­ecules in the asymmetric unit of the title compound, one of them being disordered over the methyl group. The mol­ecules are linked by weak H⋯S inter­actions into chains with graph-set motifs *C*(4) along [100] and and 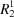
(10) rings. The Hirshfeld surface calculation suggests that the most important contribution for the crystal structure are the H⋯H inter­actions (64.20%).

## Chemical context   

The synthesis of thio­semicarbazone derivatives can be traced back to the early 1900′s (Freund & Schander, 1902 ▸). Initially, the chemically selective nucleophilic reaction with thio­semicarbazide, H_2_N—N(H)C(=S)NH_2_, was employed for the identification and characterization of aldehydes and ketones, yielding the respective thio­semicarbazone. In the 1940s it was reported that in *in vitro* assays, the thio­semicarbazone turned out to be very effective against tuberculosis. In contrast, the related oxygen-containing semicarbazones did not shown biological activity in the same assays, so that the sulfur atom in the mol­ecular structure is essential for *Mycobacterium tuberculosis* growth inhibition, a true milestone in the thio­semi­carbazone pharmacological research (Domagk *et al.*, 1946 ▸). Today, thio­semicarbazone chemistry is present across a wide range of scientific disciplines, especially inorganic coordination chemistry (Lobana *et al.*, 2009 ▸) and medicinal chemistry. For example, the synthesis, the mol­ecular docking calculation and the *in vitro* inhibition of Chikungunya virus replication by a thio­semicarbazone derivative was published in the past year (Mishra *et al.*, 2016 ▸). Thus, the crystal structure determination of thio­semicarbazone derivatives is an intensive research field, especially for biological chemistry.

## Structural commentary   

**Figure 1:**
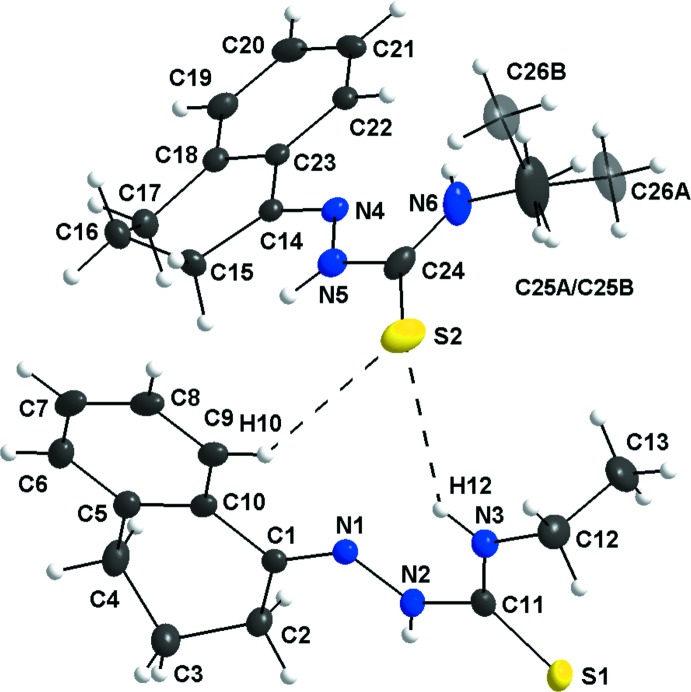
The mol­ecular structure of the title compound, with labeling and displacement ellipsoids drawn at the 40% probability level. The N3—H12⋯S2 and C9—H10⋯S2 inter­actions are drawn as dashed lines. Disordered atoms are shown with 30% transparency. The C25*A*/*B* atom is itself not disordered, but it was split using the same occupancy ratio as C26*A* and C26*B*.

The asymmetric unit shows two crystallographically independent mol­ecules, one of them being disordered over the terminal methyl group. For the disordered mol­ecule, the C25 atom was fixed with restraints and had to be split over two positions with an occupancy ratio of 0.705 (5):0.295 (5) with *A* and *B* labels. As the orientations for this *sp* ^3^-hybridized C atom are different, two possibilities for the disordered C26–atom locations are generated (Fig. 1 ▸).
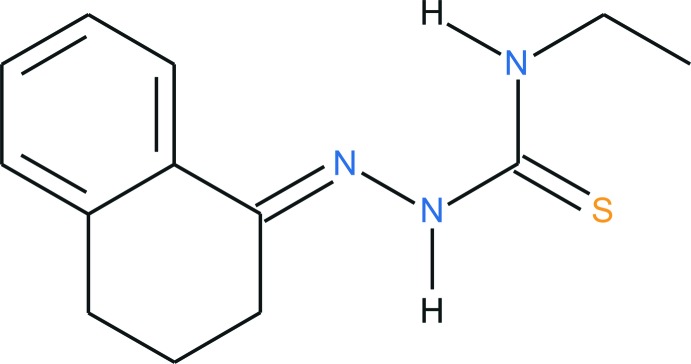


For the first mol­ecule, the C1/C2/C5/C10 atoms are essentially planar and atoms C3 and C4 deviate by 0.564 (2) and −0.142 (2) Å, respectively, from this plane. For the second, the C14/C15/C18/C23 atoms are essentially planar while atoms C16 and C17 deviate from the plane by −0.534 (2) and 0.201 (2) Å, respectively.

In addition, the N1—N2—C11—N3 and N4—N5—C24—N6 torsion angles are 9.4 (2)° and 8.3 (2)°. The dihedral angle between the tetra­lone fragments of the two mol­ecules within the asymmetric unit is 85.51 (02)°.

## Supra­molecular features and Hirshfeld surface analysis   

**Figure 2:**
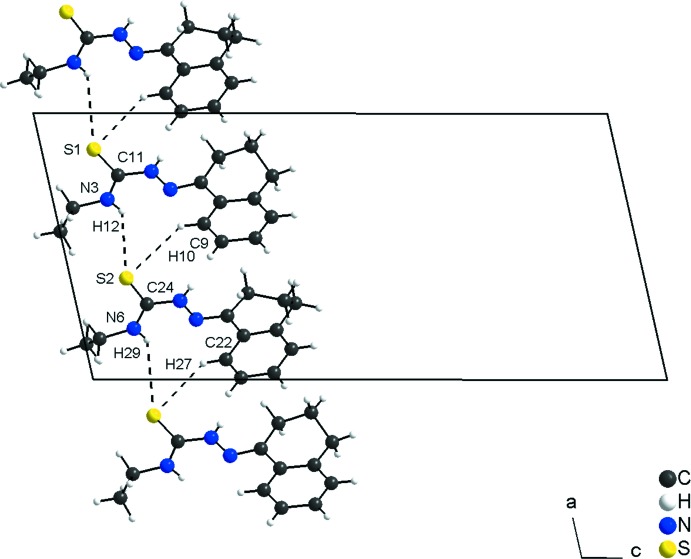
Section of the crystal structure of the title compound, showing the N—H⋯S and C—H⋯S inter­actions. The graph-set motifs for the hydrogen-bonding inter­actions in the crystal packing are *C*(4) and 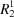
(10). The H⋯S inter­actions are shown as dashed lines and connect the mol­ecules into a chain along [100].

In the crystal, the mol­ecules are connected by weak N3—H12⋯S2 and N6—H29⋯S1^*i*^ inter­actions into chains along [100]. The S1–C11–N3–H12 and S2–C24–N6–H29 fragments are the subunits of the periodic arrangement, with graph-set motif *C*(4). In addition, the mol­ecules are linked by C9—H10⋯S2 and C22—H27⋯S1^i^ inter­actions building rings with graph-set motif 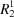
(10). The sulfur atoms are hydrogen-bond acceptors and bridge two *D*—H⋯S inter­actions (Fig. 2 ▸, Table 1 ▸).

**Figure 3:**
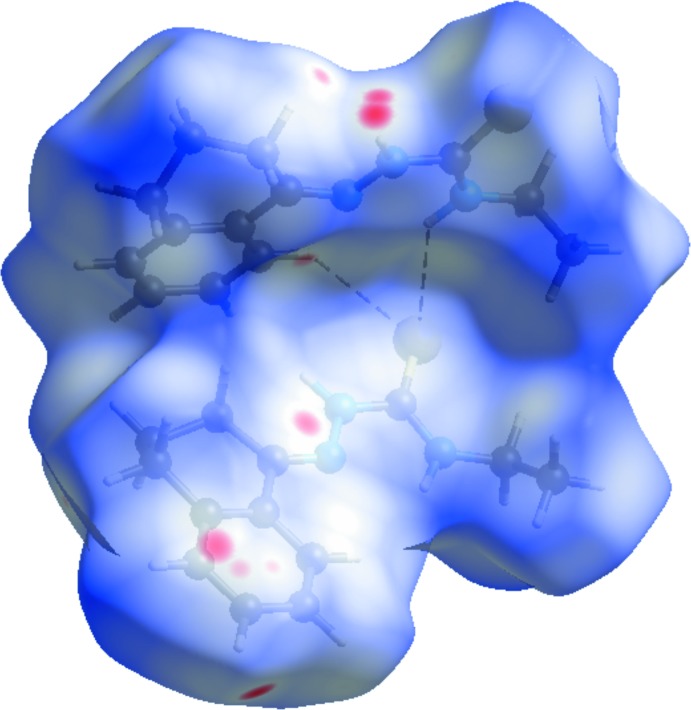
The Hirshfeld surface graphical representation (*d*_norm_) for the asymmetric unit of the title compound. The surface regions with the strongest inter­molecular inter­actions are shown in magenta. The disorder is not shown and the figure is simplified for clarity.

**Figure 4:**
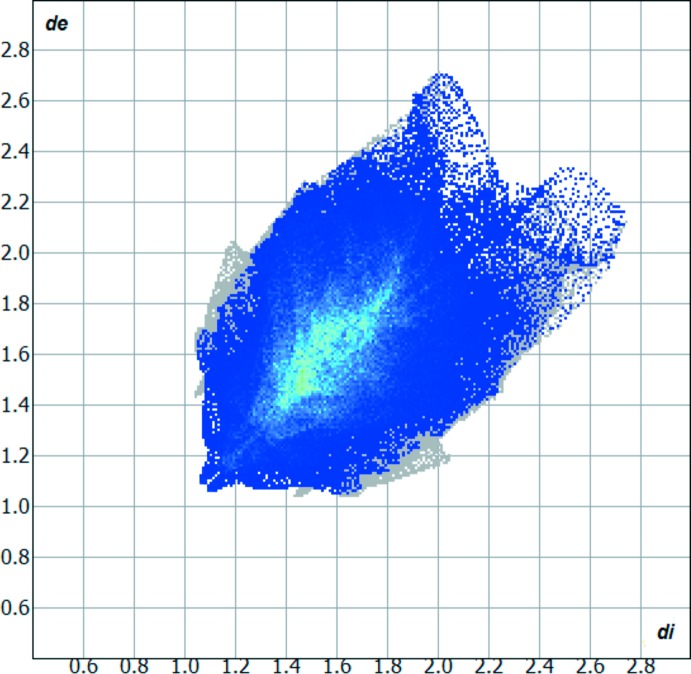
Hirshfeld surface two-dimensional fingerprint plot for the title compound showing the H⋯H contacts in detail (cyan dots). The contribution of the H⋯H inter­actions to the crystal packing amounts to 64.20%. The *d*_e_ (*y* axis) and *d*_i_ (*x* axis) values are the closest external and inter­nal distances (in Å) from given points on the Hirshfeld surface contacts. The disorder was not considered.

**Figure 5:**
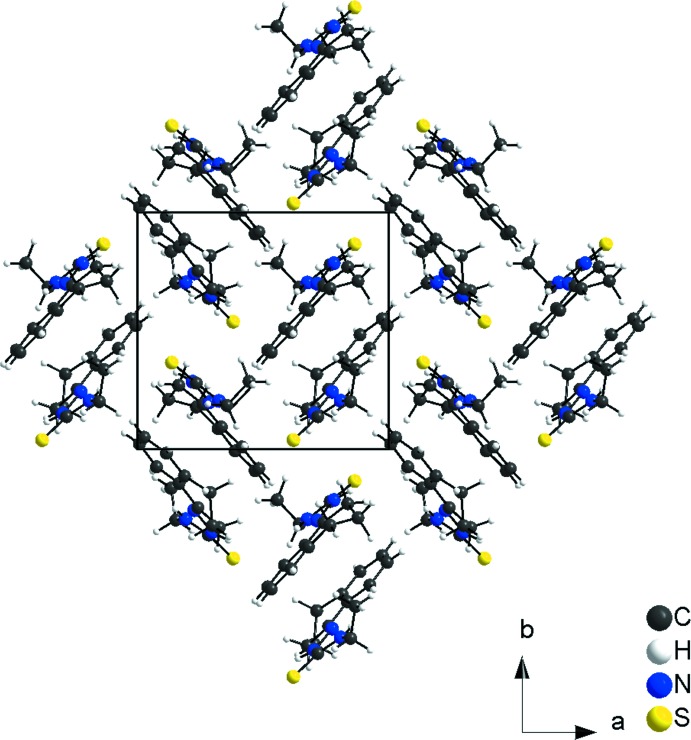
Section of the crystal structure of the title compound, in a view along the [001], showing the herringbone motif.

The Hirshfeld surface analysis (Hirshfeld, 1977 ▸) of the crystal structure suggests that the contribution of the H⋯H inter­molecular inter­actions to the crystal packing amounts to 64.20%, the H⋯S inter­actions amount to 12.60% and the H⋯C inter­actions amount to 12.00%. Other important inter­molecular contacts for the cohesion of the structure are (values given in %) are: H⋯N = 5.50, C⋯N = 3.60 and C⋯C = 2.20. For the Hirshfeld surface analysis, the disorder over the mol­ecule was not considered and the calculations were performed using the major occupancy component atoms. The graphical representation of the Hirshfeld surface (Fig. 3 ▸, represented in magenta) suggests the locations of the strongest inter­molecular contacts. The H⋯H contribution for the crystal packing is shown as a Hirshfeld surface two-dimensional fingerprint plot with cyan dots (Wolff *et al.*, 2012 ▸). The *d*_e_ (*y* axis) and *d*_i_ (*x* axis) values are the closest external and inter­nal distances (in Å) from given points on the Hirshfeld surface contacts (Fig. 4 ▸). As the most important contribution for the crystal packing is from the H⋯H inter­actions, all other inter­molecular inter­actions are relatively weak. As a consequence, the lengths of the H⋯S contacts are close to or slightly above the sum of the van der Waals radii for H and S atoms (Bondi, 1964 ▸; Rowland & Taylor, 1996 ▸). Finally, the mol­ecular packing shows a herringbone motif when viewed along [001] (Fig. 5 ▸).

## Comparison with related structures   

**Figure 6:**
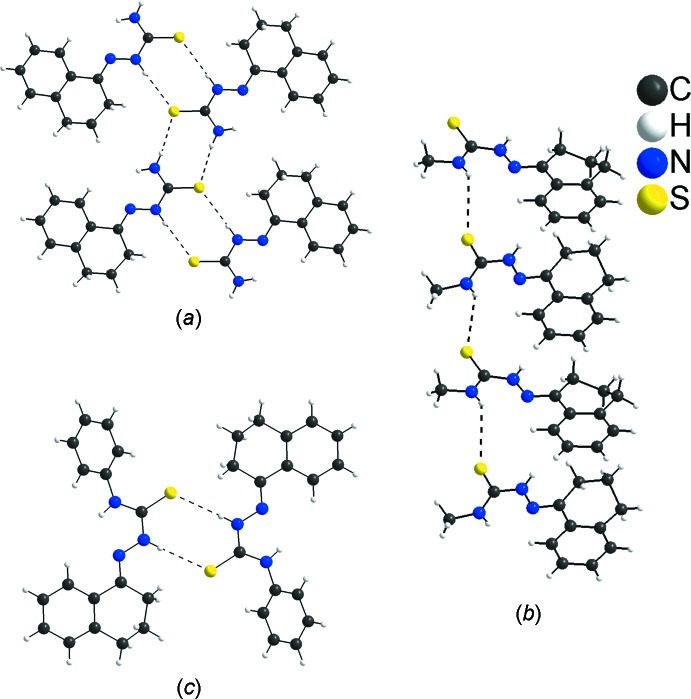
Sections of the crystal structures of 1-tetra­lone thio­semicarbazone derivatives: (*a*) 1-tetra­lone thio­semicarbazone, (*b*) 1-tetra­lone 4-methyl­thio­semicarbazone and (*c*) 1-tetra­lone 4-phenyl­thio­semicarbazone. The H⋯S inter­molecular inter­actions are shown as dashed lines. The disorder of the 1-tetra­lone 4-methyl­thio­semicarbazone mol­ecule is not shown and the figures are simplified for clarity.

**Figure 7:**
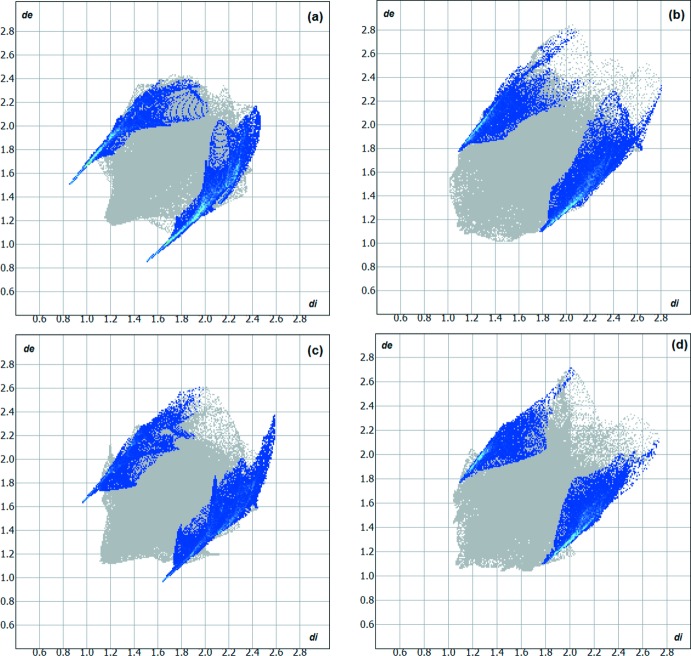
Hirshfeld surface two-dimensional fingerprint plots for the crystal structures of (*a*) 1-tetra­lone thio­semicarbazone, (*b*) 1-tetra­lone 4-methyl­thio­semicarbazone, (*c*) 1-tetra­lone 4-phenyl­lthio­semicarbazone and (*d*) 1-tetra­lone 4-ethyl­thio­semicarbazone showing the H⋯S contacts in detail (cyan dots). The H⋯S inter­actions contributions to the mol­ecular cohesion on the crystal structures amount to 19.20, 15.80, 13.00 and 12.60%, respectively.

H⋯H connections are the most important contribution for the crystal packing of 1-tetra­lone thio­semicarbazone derivatives; however, the H⋯S contacts are relevant inter­molecular inter­actions because of the possibility of forming hydrogen bonds. Therefore, *D*—H⋯S hydrogen bonding is considered in the comparison of the title compound with related structures. In the crystal structure of 2-(1,2,3,4-tetra­hydro­naph­thalen-1-yl­idene)hydrazinecarbo­thio­amide, the mol­­ecules are linked into chains by N—H⋯S hydrogen bonds (H⋯S distances = 2.45 and 2.71 Å) and the H⋯S contribution for the cohesion of the structure amounts to 19.20% (Fig. 6 ▸ *a* and 7*a*). This kind of arrangement, the one-dimensional hydrogen-bonded polymer, is possible due to the unsubstituted amine, which increases the possibilities for inter­molecular hydrogen bonding (Oliveira *et al.*, 2012 ▸). For the crystal structure of *N*-methyl-2-(1,2,3,4-tetra­hydro­naphthalen-1-yl­idene)hydrazinecarbo­thio­amide, one H atom of the amine group is substituted by one methyl group. The N—H⋯S hydrogen bonds are weaker in comparison with the first structure (H⋯S distances = 3.03 and 3.29 Å), the H⋯S contribution for the cohesion of the structure amounts to 15.80% and the dimensionality of the structure is preserved with mol­ecules linked into chains (Fig. 6 ▸ *b* and 7*b*). The disorder over the mol­ecules in the asymmetric unit was not considered and the calculations were performed using atoms of the major occupancy component (Oliveira *et al.*, 2014*a* ▸). Finally, for *N*-phenyl-2-(1,2,3,4-tetra­hydro­naphthalen-1-yl­idene)hydrazinecarbo­thio­amide, the mol­ecules are also linked by N—H⋯S hydrogen bonds, but not into hydrogen-bonded polymers (H⋯S distance = 2.70 Å). The phenyl rings linked to the amino groups change the mol­ecular arrangement due to steric effects: the mol­ecules build dimers and the H⋯S contribution to the crystal packing amounts to 13.00% (Fig. 6 ▸ *c* and 7*c*) (Oliveira *et al.*, 2014*b* ▸). For the 1-tetra­lone 4-ethyl­thio­semicarbazone reported here, the H⋯S contribution for the mol­ecular cohesion on the crystal structure amounts to 12.60% (Fig. 7 ▸ *d*). Thus, there is a relationship between the mol­ecular assembly, the geometry of the H⋯S inter­actions and their contribution to the crystal structures (Hirshfeld, 1977 ▸ and Wolff *et al.*, 2012 ▸).

## Synthesis and crystallization   

The starting materials are commercially available and were used without further purification. The synthesis of the title compound was adapted from a previously reported procedure (Freund & Schander, 1902 ▸). In a hydro­chloric acid catalysed reaction, a mixture of 1-tetra­lone (10 mmol) and 4-ethyl-3-thio­semicarbazide (10 mmol) in ethanol (80 mL) was stirred and refluxed for 4 h. After cooling and filtering, a pale-yellow solid was obtained. Colourless crystals were grown in tetra­hydro­furan by slow evaporation of the solvent.

## Refinement   

Crystal data, data collection and structure refinement details are summarized in Table 2 ▸. One of the two independent mol­ecules exhibits disorder of the methyl group. Although the secondary C atom of the ethyl substituent is not itself disordered, it was split using the same occupancy ratio as the terminal C atom to account for the different orientations of the two hydrogen atoms for the two disordered parts. The C25*A* and C25*B* atoms share the same site (EXYZ and EADP commands) and two positions will be possible for the terminal CH_*3*_–group. The C25 and C26 atoms were fixed with restraints (SADI command) and had to be split over two positions. The occupancy factor for C25*A* and C26*A* is 0.705 (5) and for C25*B* and C26*B* it is 0.295 (5). H atoms were located in difference maps but were positioned with idealized geometry and were refined with isotropic displacement parameters using a riding model (HFIX command) with *U*_iso_(H) = 1.2*U*_eq_(secondary C atoms) (C—H = 0.99 Å for aliphatic and C—H = 0.95 Å for aromatic atoms), *U*_iso_(H) = 1.5 *U*_eq_(terminal C atoms) (C—H = 0.98 Å) and *U*_iso_(H) = 1.2 *U*_eq_(N) (N—H = 0.88 Å). 

## Supplementary Material

Crystal structure: contains datablock(s) I, publication_text. DOI: 10.1107/S2056989017001311/lh5835sup1.cif

Structure factors: contains datablock(s) I. DOI: 10.1107/S2056989017001311/lh5835Isup2.hkl

Supporting information file. DOI: 10.1107/S2056989017001311/lh5835Isup3.cml

CCDC reference: 1529622

Additional supporting information:  crystallographic information; 3D view; checkCIF report

## Figures and Tables

**Table 1:** Hydrogen-bond geometry (Å, °)

*D*—H⋯*A*	*D*—H	H⋯*A*	*D*⋯*A*	*D*—H⋯*A*
N3—H12⋯S2	0.88	3.02	3.7172 (16)	138
C9—H10⋯S2	0.95	3.09	3.8835 (19)	142
N6—H29⋯S1^i^	0.88	3.31	4.002 (2)	138
C22—H27⋯S1^i^	0.95	2.98	3.7828 (17)	143

Symmetry code: (i) 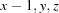.

**Table 2:** Experimental details

Crystal data	
Chemical formula	C_13_H_17_N_3_S
*M*_r_	247.36
Crystal system, space group	Monoclinic, *P*2_1_/*c*
Temperature (K)	123
*a*, *b*, *c* (Å)	11.1342 (2), 10.2330 (2), 23.3990 (5)
β (°)	102.724 (1)
*V* (Å^3^)	2600.52 (9)
*Z*	8
Radiation type	Mo *K*α
μ (mm^−1^)	0.23
Crystal size (mm)	0.35 × 0.15 × 0.05

Data collection	
Diffractometer	Nonius KappaCCD area detector
Absorption correction	Multi-scan (Blessing, 1995 ▸)
*T*_min_, *T*_max_	0.902, 0.987
No. of measured, independent and observed [*I* > 2σ(*I*)] reflections	39942, 5952, 4615
*R*_int_	0.050
(sin θ/λ)_max_ (Å^−1^)	0.649

Refinement	
*R*[*F* ^2^ > 2σ(*F* ^2^)], *wR*(*F* ^2^), *S*	0.044, 0.116, 1.02
No. of reflections	5952
No. of parameters	318
No. of restraints	1
H-atom treatment	H-atom parameters constrained
Δρ_max_, Δρ_min_ (e Å^−3^)	0.48, −0.41

Computer programs: *COLLECT* (Nonius, 1998 ▸), *HKL*, *DENZO* and *SCALEPACK* (Otwinowski & Minor, 1997 ▸), *SUPERFLIP* (Palatinus & Chapuis, 2007 ▸), *SHELXL97* (Sheldrick, 2008 ▸), *DIAMOND* (Brandenburg, 2006 ▸), *Crystal Explorer* (Wolff *et al.*, 2012 ▸), *publCIF* (Westrip, 2010 ▸) and *enCIFer* (Allen *et al.*, 2004 ▸).

## supplementary crystallographic information

### Crystal data

**Table:**

C_13_H_17_N_3_S	*F*(000) = 1056
*M**_r_* = 247.36	*D*_x_ = 1.264 Mg m^−^^3^
Monoclinic, *P*2_1_/*c*	Mo *K*α radiation, λ = 0.71073 Å
Hall symbol: -P 2ybc	Cell parameters from 42401 reflections
*a* = 11.1342 (2) Å	θ = 2.9–27.5°
*b* = 10.2330 (2) Å	µ = 0.23 mm^−^^1^
*c* = 23.3990 (5) Å	*T* = 123 K
β = 102.724 (1)°	Block, colourless
*V* = 2600.52 (9) Å^3^	0.35 × 0.15 × 0.05 mm
*Z* = 8	

### Data collection

**Table:**

Nonius KappaCCD area detector diffractometer	5952 independent reflections
Radiation source: fine-focus sealed tube, Nonius Kappa CCD	4615 reflections with *I* > 2σ(*I*)
Graphite monochromator	*R*_int_ = 0.050
Detector resolution: 9 pixels mm^-1^	θ_max_ = 27.5°, θ_min_ = 3.0°
CCD rotation images, thick slices scans	*h* = −14→14
Absorption correction: multi-scan (Blessing, 1995)	*k* = −13→13
*T*_min_ = 0.902, *T*_max_ = 0.987	*l* = −30→28
39942 measured reflections	

### Refinement

**Table:**

Refinement on *F*^2^	Primary atom site location: structure-invariant direct methods
Least-squares matrix: full	Secondary atom site location: difference Fourier map
*R*[*F*^2^ > 2σ(*F*^2^)] = 0.044	Hydrogen site location: inferred from neighbouring sites
*wR*(*F*^2^) = 0.116	H-atom parameters constrained
*S* = 1.02	*w* = 1/[σ^2^(*F*_o_^2^) + (0.0524*P*)^2^ + 1.4032*P*] where *P* = (*F*_o_^2^ + 2*F*_c_^2^)/3
5952 reflections	(Δ/σ)_max_ < 0.001
318 parameters	Δρ_max_ = 0.48 e Å^−^^3^
1 restraint	Δρ_min_ = −0.41 e Å^−^^3^

### Special details

**Table:**

Geometry. All esds (except the esd in the dihedral angle between two l.s. planes) are estimated using the full covariance matrix. The cell esds are taken into account individually in the estimation of esds in distances, angles and torsion angles; correlations between esds in cell parameters are only used when they are defined by crystal symmetry. An approximate (isotropic) treatment of cell esds is used for estimating esds involving l.s. planes.
Refinement. Refinement of F^2^ against ALL reflections. The weighted R-factor wR and goodness of fit S are based on F^2^, conventional R-factors R are based on F, with F set to zero for negative F^2^. The threshold expression of F^2^ > 2sigma(F^2^) is used only for calculating R-factors(gt) etc. and is not relevant to the choice of reflections for refinement. R-factors based on F^2^ are statistically about twice as large as those based on F, and R- factors based on ALL data will be even larger.

### Fractional atomic coordinates and isotropic or equivalent isotropic displacement parameters (Å^2^)

**Table:**

	*x*	*y*	*z*	*U*_iso_*/*U*_eq_	Occ. (<1)
C1	0.74029 (14)	0.81156 (15)	0.26519 (7)	0.0204 (3)	
C2	0.83649 (16)	0.73161 (17)	0.30579 (7)	0.0259 (4)	
H1	0.9095	0.7229	0.2884	0.031*	
H2	0.8034	0.6429	0.3092	0.031*	
C3	0.87661 (18)	0.7906 (2)	0.36697 (8)	0.0338 (4)	
H3	0.9301	0.7279	0.3931	0.041*	
H4	0.9248	0.8711	0.3650	0.041*	
C4	0.76544 (19)	0.8229 (2)	0.39190 (8)	0.0356 (4)	
H5	0.7211	0.7413	0.3970	0.043*	
H6	0.7930	0.8641	0.4309	0.043*	
C5	0.67946 (16)	0.91465 (17)	0.35168 (8)	0.0271 (4)	
C6	0.61121 (17)	1.00840 (19)	0.37437 (9)	0.0331 (4)	
H7	0.6197	1.0146	0.4156	0.040*	
C7	0.53164 (17)	1.09221 (18)	0.33808 (9)	0.0338 (4)	
H8	0.4864	1.1556	0.3543	0.041*	
C8	0.51821 (16)	1.08314 (17)	0.27784 (9)	0.0302 (4)	
H9	0.4627	1.1397	0.2527	0.036*	
C9	0.58507 (15)	0.99229 (16)	0.25425 (8)	0.0255 (4)	
H10	0.5758	0.9872	0.2130	0.031*	
C10	0.66681 (15)	0.90726 (16)	0.29086 (7)	0.0223 (3)	
C11	0.76692 (15)	0.72188 (16)	0.12327 (7)	0.0233 (3)	
C12	0.64454 (19)	0.8165 (2)	0.03189 (8)	0.0359 (4)	
H13	0.7207	0.8130	0.0166	0.043*	
H14	0.6077	0.9042	0.0231	0.043*	
C13	0.5557 (2)	0.7151 (3)	0.00106 (9)	0.0498 (6)	
H15	0.5906	0.6278	0.0106	0.075*	
H16	0.5407	0.7290	−0.0414	0.075*	
H17	0.4779	0.7224	0.0138	0.075*	
C14	0.23948 (14)	0.74836 (15)	0.25873 (7)	0.0195 (3)	
C15	0.32438 (16)	0.83155 (17)	0.30316 (7)	0.0261 (4)	
H18	0.3173	0.9235	0.2897	0.031*	
H19	0.4103	0.8032	0.3055	0.031*	
C16	0.29737 (17)	0.82471 (18)	0.36413 (8)	0.0294 (4)	
H20	0.3641	0.8686	0.3926	0.035*	
H21	0.2193	0.8709	0.3643	0.035*	
C17	0.28738 (16)	0.68370 (18)	0.38210 (7)	0.0280 (4)	
H22	0.3667	0.6387	0.3837	0.034*	
H23	0.2694	0.6804	0.4217	0.034*	
C18	0.18651 (15)	0.61472 (16)	0.33905 (7)	0.0221 (3)	
C19	0.11481 (16)	0.51819 (17)	0.35743 (8)	0.0269 (4)	
H24	0.1274	0.4984	0.3980	0.032*	
C20	0.02604 (16)	0.45102 (17)	0.31785 (8)	0.0285 (4)	
H25	−0.0221	0.3858	0.3311	0.034*	
C21	0.00738 (15)	0.47942 (17)	0.25843 (8)	0.0265 (4)	
H26	−0.0525	0.4322	0.2310	0.032*	
C22	0.07550 (15)	0.57592 (16)	0.23918 (7)	0.0227 (3)	
H27	0.0615	0.5955	0.1986	0.027*	
C23	0.16531 (14)	0.64538 (15)	0.27924 (7)	0.0192 (3)	
C24	0.28275 (18)	0.86401 (18)	0.12330 (8)	0.0309 (4)	
C25A	0.1525 (3)	0.8000 (3)	0.02723 (10)	0.0733 (9)	0.705 (5)
H30	0.2162	0.8448	0.0108	0.088*	0.705 (5)
H31	0.0751	0.8504	0.0157	0.088*	0.705 (5)
C26A	0.1331 (4)	0.6658 (4)	0.00242 (14)	0.0625 (12)	0.705 (5)
H34	0.1060	0.6709	−0.0403	0.094*	0.705 (5)
H35	0.0700	0.6213	0.0186	0.094*	0.705 (5)
H36	0.2104	0.6166	0.0127	0.094*	0.705 (5)
C25B	0.1525 (3)	0.8000 (3)	0.02723 (10)	0.0733 (9)	0.295 (5)
H32	0.1167	0.7150	0.0120	0.088*	0.295 (5)
H33	0.2232	0.8199	0.0093	0.088*	0.295 (5)
C26B	0.0497 (8)	0.9155 (8)	0.0132 (3)	0.053 (3)	0.295 (5)
H37	0.0185	0.9224	−0.0292	0.080*	0.295 (5)
H38	0.0871	0.9987	0.0285	0.080*	0.295 (5)
H39	−0.0185	0.8948	0.0320	0.080*	0.295 (5)
N1	0.71417 (12)	0.80254 (13)	0.20897 (6)	0.0214 (3)	
N2	0.78106 (13)	0.71646 (13)	0.18298 (6)	0.0231 (3)	
H11	0.8314	0.6596	0.2041	0.028*	
N3	0.67615 (13)	0.79765 (14)	0.09517 (6)	0.0260 (3)	
H12	0.6320	0.8394	0.1162	0.031*	
N4	0.22574 (12)	0.75948 (13)	0.20292 (6)	0.0217 (3)	
N5	0.29620 (13)	0.85150 (14)	0.18236 (6)	0.0262 (3)	
H28	0.3485	0.9009	0.2067	0.031*	
N6	0.19103 (18)	0.79705 (17)	0.09097 (7)	0.0409 (4)	
H29	0.1492	0.7457	0.1097	0.049*	
S1	0.86369 (4)	0.63602 (5)	0.091362 (19)	0.03122 (13)	
S2	0.38000 (5)	0.96310 (6)	0.09740 (2)	0.04411 (16)	

### Atomic displacement parameters (Å^2^)

**Table:**

	*U*^11^	*U*^22^	*U*^33^	*U*^12^	*U*^13^	*U*^23^
C1	0.0202 (8)	0.0188 (8)	0.0229 (8)	−0.0027 (6)	0.0061 (6)	0.0006 (6)
C2	0.0298 (9)	0.0260 (9)	0.0223 (8)	0.0062 (7)	0.0067 (7)	0.0012 (7)
C3	0.0368 (11)	0.0373 (11)	0.0252 (9)	0.0077 (8)	0.0022 (8)	0.0006 (8)
C4	0.0457 (12)	0.0378 (11)	0.0242 (9)	0.0063 (9)	0.0094 (8)	−0.0017 (8)
C5	0.0264 (9)	0.0261 (9)	0.0302 (9)	−0.0026 (7)	0.0089 (7)	−0.0033 (7)
C6	0.0333 (10)	0.0349 (10)	0.0341 (10)	−0.0053 (8)	0.0134 (8)	−0.0111 (8)
C7	0.0260 (9)	0.0250 (9)	0.0541 (12)	−0.0028 (7)	0.0169 (9)	−0.0104 (9)
C8	0.0209 (8)	0.0221 (9)	0.0487 (11)	−0.0008 (7)	0.0099 (8)	0.0002 (8)
C9	0.0212 (8)	0.0221 (8)	0.0344 (9)	−0.0026 (7)	0.0087 (7)	0.0014 (7)
C10	0.0199 (8)	0.0199 (8)	0.0287 (9)	−0.0030 (6)	0.0089 (7)	−0.0007 (7)
C11	0.0260 (9)	0.0228 (8)	0.0206 (8)	−0.0047 (7)	0.0044 (7)	−0.0001 (6)
C12	0.0400 (11)	0.0446 (12)	0.0222 (9)	0.0059 (9)	0.0048 (8)	0.0087 (8)
C13	0.0402 (12)	0.0742 (17)	0.0295 (11)	−0.0016 (11)	−0.0039 (9)	−0.0083 (11)
C14	0.0180 (7)	0.0202 (8)	0.0210 (8)	0.0027 (6)	0.0057 (6)	0.0000 (6)
C15	0.0244 (9)	0.0262 (9)	0.0270 (9)	−0.0068 (7)	0.0041 (7)	−0.0011 (7)
C16	0.0291 (9)	0.0308 (9)	0.0267 (9)	−0.0023 (7)	0.0025 (7)	−0.0054 (7)
C17	0.0293 (9)	0.0330 (10)	0.0209 (8)	−0.0013 (7)	0.0038 (7)	−0.0001 (7)
C18	0.0210 (8)	0.0231 (8)	0.0230 (8)	0.0035 (6)	0.0064 (6)	0.0012 (6)
C19	0.0263 (9)	0.0283 (9)	0.0285 (9)	0.0049 (7)	0.0108 (7)	0.0071 (7)
C20	0.0224 (9)	0.0221 (8)	0.0433 (11)	0.0013 (7)	0.0123 (8)	0.0068 (7)
C21	0.0186 (8)	0.0215 (8)	0.0386 (10)	−0.0012 (6)	0.0045 (7)	−0.0034 (7)
C22	0.0187 (8)	0.0239 (8)	0.0252 (8)	0.0030 (6)	0.0040 (6)	−0.0021 (7)
C23	0.0169 (7)	0.0185 (8)	0.0230 (8)	0.0034 (6)	0.0060 (6)	0.0000 (6)
C24	0.0393 (11)	0.0284 (9)	0.0293 (9)	0.0125 (8)	0.0172 (8)	0.0089 (8)
C25A	0.126 (3)	0.0721 (19)	0.0224 (11)	−0.0116 (18)	0.0169 (14)	0.0022 (11)
C26A	0.082 (3)	0.063 (2)	0.0323 (17)	−0.003 (2)	−0.0089 (17)	−0.0075 (16)
C25B	0.126 (3)	0.0721 (19)	0.0224 (11)	−0.0116 (18)	0.0169 (14)	0.0022 (11)
C26B	0.066 (6)	0.064 (6)	0.025 (4)	0.011 (4)	−0.001 (3)	0.008 (3)
N1	0.0218 (7)	0.0190 (7)	0.0239 (7)	−0.0011 (5)	0.0062 (5)	−0.0004 (5)
N2	0.0270 (7)	0.0211 (7)	0.0208 (7)	0.0036 (6)	0.0045 (6)	0.0009 (5)
N3	0.0275 (8)	0.0282 (8)	0.0217 (7)	0.0006 (6)	0.0044 (6)	0.0013 (6)
N4	0.0222 (7)	0.0209 (7)	0.0238 (7)	0.0027 (5)	0.0091 (6)	0.0033 (5)
N5	0.0284 (8)	0.0263 (8)	0.0257 (7)	0.0002 (6)	0.0099 (6)	0.0053 (6)
N6	0.0652 (12)	0.0371 (9)	0.0223 (8)	−0.0036 (9)	0.0137 (8)	0.0017 (7)
S1	0.0346 (3)	0.0357 (3)	0.0240 (2)	0.0030 (2)	0.00759 (18)	−0.00517 (18)
S2	0.0437 (3)	0.0503 (3)	0.0441 (3)	0.0090 (2)	0.0224 (2)	0.0260 (2)

### Geometric parameters (Å, º)

**Table:**

C1—N1	1.287 (2)	C15—H19	0.9900
C1—C10	1.485 (2)	C16—C17	1.514 (3)
C1—C2	1.507 (2)	C16—H20	0.9900
C2—C3	1.527 (2)	C16—H21	0.9900
C2—H1	0.9900	C17—C18	1.509 (2)
C2—H2	0.9900	C17—H22	0.9900
C3—C4	1.516 (3)	C17—H23	0.9900
C3—H3	0.9900	C18—C19	1.396 (2)
C3—H4	0.9900	C18—C23	1.403 (2)
C4—C5	1.513 (3)	C19—C20	1.380 (3)
C4—H5	0.9900	C19—H24	0.9500
C4—H6	0.9900	C20—C21	1.391 (3)
C5—C6	1.399 (2)	C20—H25	0.9500
C5—C10	1.401 (2)	C21—C22	1.380 (2)
C6—C7	1.382 (3)	C21—H26	0.9500
C6—H7	0.9500	C22—C23	1.404 (2)
C7—C8	1.387 (3)	C22—H27	0.9500
C7—H8	0.9500	C24—N6	1.321 (3)
C8—C9	1.380 (2)	C24—N5	1.363 (2)
C8—H9	0.9500	C24—S2	1.6904 (19)
C9—C10	1.406 (2)	C25A—N6	1.458 (3)
C9—H10	0.9500	C25A—C26A	1.489 (4)
C11—N3	1.327 (2)	C25A—H30	0.9900
C11—N2	1.372 (2)	C25A—H31	0.9900
C11—S1	1.6866 (17)	C26A—H34	0.9800
C12—N3	1.457 (2)	C26A—H35	0.9800
C12—C13	1.503 (3)	C26A—H36	0.9800
C12—H13	0.9900	C26B—H37	0.9800
C12—H14	0.9900	C26B—H38	0.9800
C13—H15	0.9800	C26B—H39	0.9800
C13—H16	0.9800	N1—N2	1.3785 (18)
C13—H17	0.9800	N2—H11	0.8800
C14—N4	1.286 (2)	N3—H12	0.8800
C14—C23	1.482 (2)	N4—N5	1.3785 (19)
C14—C15	1.505 (2)	N5—H28	0.8800
C15—C16	1.523 (2)	N6—H29	0.8800
C15—H18	0.9900		
			
N1—C1—C10	116.11 (14)	C17—C16—C15	110.22 (15)
N1—C1—C2	125.09 (14)	C17—C16—H20	109.6
C10—C1—C2	118.78 (14)	C15—C16—H20	109.6
C1—C2—C3	113.38 (14)	C17—C16—H21	109.6
C1—C2—H1	108.9	C15—C16—H21	109.6
C3—C2—H1	108.9	H20—C16—H21	108.1
C1—C2—H2	108.9	C18—C17—C16	110.49 (14)
C3—C2—H2	108.9	C18—C17—H22	109.6
H1—C2—H2	107.7	C16—C17—H22	109.6
C4—C3—C2	110.59 (16)	C18—C17—H23	109.6
C4—C3—H3	109.5	C16—C17—H23	109.6
C2—C3—H3	109.5	H22—C17—H23	108.1
C4—C3—H4	109.5	C19—C18—C23	118.92 (15)
C2—C3—H4	109.5	C19—C18—C17	121.17 (15)
H3—C3—H4	108.1	C23—C18—C17	119.90 (15)
C5—C4—C3	110.73 (15)	C20—C19—C18	121.30 (16)
C5—C4—H5	109.5	C20—C19—H24	119.4
C3—C4—H5	109.5	C18—C19—H24	119.4
C5—C4—H6	109.5	C19—C20—C21	119.62 (16)
C3—C4—H6	109.5	C19—C20—H25	120.2
H5—C4—H6	108.1	C21—C20—H25	120.2
C6—C5—C10	118.75 (17)	C22—C21—C20	120.27 (16)
C6—C5—C4	120.79 (16)	C22—C21—H26	119.9
C10—C5—C4	120.46 (15)	C20—C21—H26	119.9
C7—C6—C5	121.38 (18)	C21—C22—C23	120.42 (16)
C7—C6—H7	119.3	C21—C22—H27	119.8
C5—C6—H7	119.3	C23—C22—H27	119.8
C6—C7—C8	119.59 (17)	C18—C23—C22	119.44 (15)
C6—C7—H8	120.2	C18—C23—C14	119.92 (14)
C8—C7—H8	120.2	C22—C23—C14	120.63 (14)
C9—C8—C7	120.30 (18)	N6—C24—N5	115.50 (16)
C9—C8—H9	119.8	N6—C24—S2	125.54 (14)
C7—C8—H9	119.8	N5—C24—S2	118.95 (15)
C8—C9—C10	120.47 (17)	N6—C25A—C26A	111.4 (2)
C8—C9—H10	119.8	N6—C25A—H30	109.4
C10—C9—H10	119.8	C26A—C25A—H30	109.4
C5—C10—C9	119.51 (15)	N6—C25A—H31	109.4
C5—C10—C1	120.35 (15)	C26A—C25A—H31	109.4
C9—C10—C1	120.14 (15)	H30—C25A—H31	108.0
N3—C11—N2	115.60 (15)	C25A—C26A—H34	109.5
N3—C11—S1	125.16 (13)	C25A—C26A—H35	109.5
N2—C11—S1	119.24 (13)	H34—C26A—H35	109.5
N3—C12—C13	112.49 (17)	C25A—C26A—H36	109.5
N3—C12—H13	109.1	H34—C26A—H36	109.5
C13—C12—H13	109.1	H35—C26A—H36	109.5
N3—C12—H14	109.1	H37—C26B—H38	109.5
C13—C12—H14	109.1	H37—C26B—H39	109.5
H13—C12—H14	107.8	H38—C26B—H39	109.5
C12—C13—H15	109.5	C1—N1—N2	118.32 (14)
C12—C13—H16	109.5	C11—N2—N1	118.12 (14)
H15—C13—H16	109.5	C11—N2—H11	120.9
C12—C13—H17	109.5	N1—N2—H11	120.9
H15—C13—H17	109.5	C11—N3—C12	124.58 (15)
H16—C13—H17	109.5	C11—N3—H12	117.7
N4—C14—C23	116.20 (14)	C12—N3—H12	117.7
N4—C14—C15	124.57 (15)	C14—N4—N5	117.74 (14)
C23—C14—C15	119.23 (14)	C24—N5—N4	118.31 (15)
C14—C15—C16	113.42 (14)	C24—N5—H28	120.8
C14—C15—H18	108.9	N4—N5—H28	120.8
C16—C15—H18	108.9	C24—N6—C25A	126.23 (19)
C14—C15—H19	108.9	C24—N6—H29	116.9
C16—C15—H19	108.9	C25A—N6—H29	116.9
H18—C15—H19	107.7		
			
N1—C1—C2—C3	161.54 (16)	C18—C19—C20—C21	0.1 (3)
C10—C1—C2—C3	−19.7 (2)	C19—C20—C21—C22	−1.2 (3)
C1—C2—C3—C4	50.9 (2)	C20—C21—C22—C23	0.8 (2)
C2—C3—C4—C5	−57.0 (2)	C19—C18—C23—C22	−1.9 (2)
C3—C4—C5—C6	−146.70 (17)	C17—C18—C23—C22	176.74 (15)
C3—C4—C5—C10	33.3 (2)	C19—C18—C23—C14	179.44 (14)
C10—C5—C6—C7	0.5 (3)	C17—C18—C23—C14	−1.9 (2)
C4—C5—C6—C7	−179.51 (18)	C21—C22—C23—C18	0.8 (2)
C5—C6—C7—C8	0.4 (3)	C21—C22—C23—C14	179.48 (14)
C6—C7—C8—C9	−0.9 (3)	N4—C14—C23—C18	171.47 (14)
C7—C8—C9—C10	0.5 (3)	C15—C14—C23—C18	−8.4 (2)
C6—C5—C10—C9	−0.9 (2)	N4—C14—C23—C22	−7.2 (2)
C4—C5—C10—C9	179.10 (16)	C15—C14—C23—C22	172.93 (15)
C6—C5—C10—C1	178.29 (15)	C10—C1—N1—N2	179.05 (13)
C4—C5—C10—C1	−1.7 (2)	C2—C1—N1—N2	−2.1 (2)
C8—C9—C10—C5	0.4 (2)	N3—C11—N2—N1	−9.4 (2)
C8—C9—C10—C1	−178.77 (15)	S1—C11—N2—N1	170.01 (11)
N1—C1—C10—C5	173.40 (15)	C1—N1—N2—C11	−169.76 (14)
C2—C1—C10—C5	−5.5 (2)	N2—C11—N3—C12	−179.47 (16)
N1—C1—C10—C9	−7.4 (2)	S1—C11—N3—C12	1.2 (3)
C2—C1—C10—C9	173.72 (15)	C13—C12—N3—C11	87.5 (2)
N4—C14—C15—C16	163.89 (16)	C23—C14—N4—N5	−177.99 (13)
C23—C14—C15—C16	−16.2 (2)	C15—C14—N4—N5	1.9 (2)
C14—C15—C16—C17	49.6 (2)	N6—C24—N5—N4	8.3 (2)
C15—C16—C17—C18	−58.95 (19)	S2—C24—N5—N4	−172.76 (11)
C16—C17—C18—C19	−145.52 (16)	C14—N4—N5—C24	179.83 (15)
C16—C17—C18—C23	35.9 (2)	N5—C24—N6—C25A	175.9 (2)
C23—C18—C19—C20	1.4 (2)	S2—C24—N6—C25A	−3.0 (3)
C17—C18—C19—C20	−177.19 (16)	C26A—C25A—N6—C24	133.2 (3)

### Hydrogen-bond geometry (Å, º)

**Table:**

*D*—H···*A*	*D*—H	H···*A*	*D*···*A*	*D*—H···*A*
N3—H12···S2	0.88	3.02	3.7172 (16)	138
C9—H10···S2	0.95	3.09	3.8835 (19)	142
N6—H29···S1^i^	0.88	3.31	4.002 (2)	138
C22—H27···S1^i^	0.95	2.98	3.7828 (17)	143

Symmetry code: (i) *x*−1, *y*, *z*.
